# Chromosome alterations and E-cadherin gene mutations in human lobular breast cancer

**DOI:** 10.1038/sj.bjc.6690815

**Published:** 1999-12

**Authors:** C Huiping, J R Sigurgeirsdottir, J G Jonasson, G Eiriksdottir, J T Johannsdottir, V Egilsson, S Ingvarsson

**Affiliations:** Department of Pathology, National University Hospital, PO Box 1465, IS-121 Reykjavik, Iceland

**Keywords:** E-cadherin, lobular breast cancer, mutations, LOH, tumour suppressor gene

## Abstract

We have studied a set of 40 human lobular breast cancers for loss of heterozygosity (LOH) at various chromosome locations and for mutations in the coding region plus flanking intron sequences of the E-cadherin gene. We found a high frequency of LOH (100%, 31/31) at 16q21–q22.1. A significantly higher level of LOH was detected in ductal breast tumours at chromosome arms 1p, 3p, 9p, 11q, 13q and 18q compared to lobular breast tumours. Furthermore, we found a significant association between LOH at 16 q containing the E-cadherin locus and lobular histological type. Six different somatic mutations were detected in the E-cadherin gene, of which three were insertions, two deletions and one splice site mutation. Mutations were found in combination with LOH of the wild type E-cadherin locus and loss of or reduced E-cadherin expression detected by immunohistochemistry. The mutations described here have not previously been reported. We compared LOH at different chromosome regions with E-cadherin gene mutations and found a significant association between LOH at 13 q and E-cadherin gene mutations. A significant association was also detected between LOH at 13q and LOH at 7q and 11q. Moreover, we found a significant association between LOH at 3 p and high S phase, LOH at 9p and low ER and PgR content, LOH at 17p and aneuploidy. We conclude that LOH at 16q is the most frequent chromosome alteration and E-cadherin is a typical tumour suppressor gene in lobular breast cancer. © 1999 Cancer Research Campaign


in human lobular breast cancer **81**(7), 1103–1110
© 1999 Cancer Research Campaign
Article no. bjoc.1999.0815

### Chromosome alterations and E-cadherin gene mutations in human lobular breast cancer

**C Huiping, JR Sigurgeirsdottir, JG Jonasson, G Eiriksdottir, JT Johannsdottir, V Egilsson and S Ingvarsson**

Department of Pathology, National University Hospital, PO Box 1465, IS-121 Reykjavik, Iceland

**Summary** We have studied a set of 40 human lobular breast cancers for loss of heterozygosity (LOH) at various chromosome locations and for mutations in the coding region plus flanking intron sequences of the E-cadherin gene. We found a high frequency of LOH (100%, 31/31) at 16q21–q22.1. A significantly higher level of LOH was detected in ductal breast tumours at chromosome arms 1p, 3p, 9p, 11q, 13q and 18q compared to lobular breast tumours. Furthermore, we found a significant association between LOH at 16 q containing the E-cadherin locus and lobular histological type. Six different somatic mutations were detected in the E-cadherin gene, of which three were insertions, two deletions and one splice site mutation. Mutations were found in combination with LOH of the wild type E-cadherin locus and loss of or reduced E-cadherin expression detected by immunohistochemistry. The mutations described here have not previously been reported. We compared LOH at different chromosome regions with E-cadherin gene mutations and found a significant association between LOH at 13 q and E-cadherin gene mutations. A significant association was also detected between LOH at 13q and LOH at 7q and 11q. Moreover, we found a significant association between LOH at 3 p and high S phase, LOH at 9p and low ER and PgR content, LOH at 17p and aneuploidy. We conclude that LOH at 16q is the most frequent chromosome alteration and E-cadherin is a typical tumour suppressor gene in lobular breast cancer. © 1999 Cancer Research Campaign

**Keywords:** E-cadherin; lobular breast cancer; mutations; LOH; tumour suppressor gene

Loss of heterozygosity (LOH) has been studied at many chromosome regions in sporadic breast cancers and at least 15 different chromosome arms have shown frequent LOH (Ingvarsson, 1999). LOH at 16q is one of the most frequently occurring genetic events in sporadic breast cancer (~67% of the informative cases), indicating the presence of one or more tumour suppressor genes at 16q (Tsuda et al, 1994; Skirnisdottir et al, 1995).

The E-cadherin gene is one of the candidate tumour suppressor genes at 16q22.1, which is one of the smallest deletion regions at 16q (Cleton-Jansen et al, 1994). E-cadherin is expressed on the cell surface in most epithelial tissues (Takeichi, 1990). The transmembrane molecule E-cadherin is considered to be one of the key molecules for the formation of the intercellular junctional complex and for the establishment of cell polarization (Gumbiner et al, 1988). The cytoplasmic tail of E-cadherin is linked via catenins to the actin cytoskeleton (Cowin, 1994), whereas the extracellular domain is involved in a molecular zipper mediating cell–cell adhesion (Shapiro et al, 1995). E-cadherin showed an important invasion suppressor activity in vitro (Frixen et al, 1991). Reduced E-cadherin expression is associated with invasiveness in breast cancer (Siitonen et al, 1996). Activation of E-cadherin can cause growth retardation of tumour cells (Navarro et al, 1991; St Croix et al, 1998). Mutations of the gene E-cadherin have been reported so far in gastric carcinomas (Becker et al, 1993, 1994), gastric carcinoma cell lines (Oda et al, 1994), cancers of the endometrium and ovarium (Risinger et al, 1994), breast cancer cell lines (Hiraguri et al, 1998) and lobular breast cancers (Kanai et al, 1994; Berx et al, 1995*a*, 1996). Furthermore, germline mutations in the E-cadherin gene have been described in familial gastric cancer (Gayther et al, 1998; Guilford et al, 1998). These results strongly suggest that the E-cadherin gene is a tumour suppressor gene.

By screening four exons (5, 6, 7 and 8) of the E-cadherin gene, Kanai et al (1994) found that two (10%) of 20 lobular breast carcinomas showed point mutations. Berx et al (1996) reported 27 E-cadherin mutations detected from a majority of 16 exons of E-cadherin gene in a series of 48 lobular breast carcinomas. These mutations were obviously scattered over the whole E-cadherin gene, particularly in the exons encoding the extracellular domain (Kanai et al, 1994; Berx et al, 1996). Interestingly, no mutations were identified in 50 breast cancers of other histological subtypes by Berx et al (1996).

In order to study the difference of genetic alterations in lobular and ductal types of breast tumours we analysed LOH at 1p, 3p, 6q, 7q, 9p, 11q, 13q, 16q, 17q, 18q and 20q. We also investigated the association between LOH at 16q and lobular histological type in order to understand the involvement of 16q in the aetiology of lobular breast carcinomas. Furthermore, we screened a set of lobular breast tumours in an attempt to find new E-cadherin mutations.

### MATERIALS AND METHODS

### Patients and tumour material

All 40 patients were diagnosed histologically as lobular breast cancers at Department of Pathology of the University Hospital of Iceland. Primary breast carcinoma tissue was obtained on the day of surgery. Blood samples from the patients were collected in EDTA and if not processed immediately the tumour tissue and

*Received 26 January 1999*
*Revised 5 May 1999*
*Accepted 13 May 1999*

*Correspondence to:* S Ingvarsson

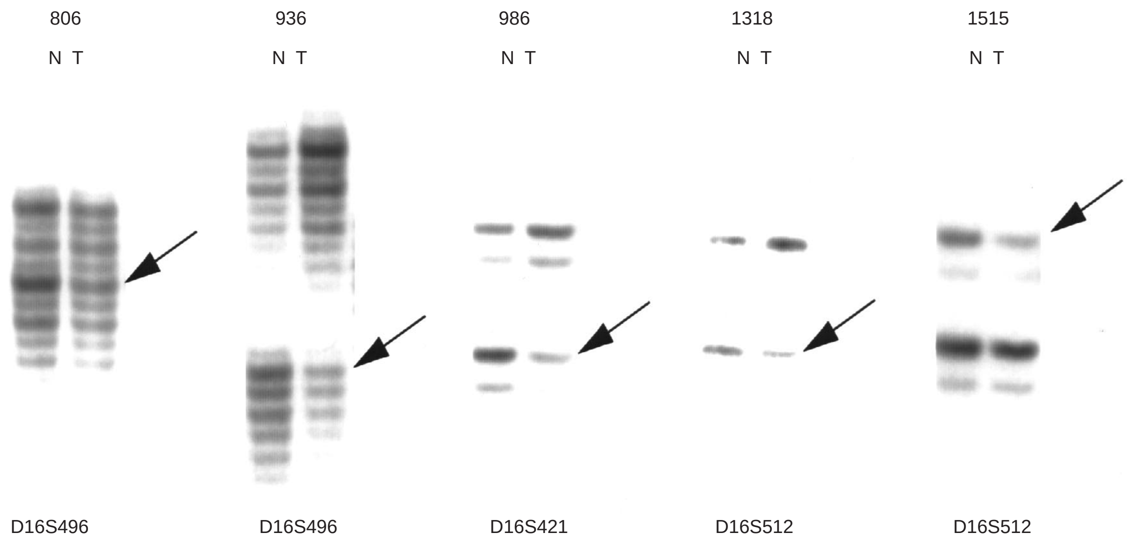

**Figure 1** LOH in matched normal (N) and tumour (T) tissues from five lobular breast cancer patients with E-cadherin gene mutations. Case numbers are shown at the top. Symbols at the bottom indicate the markers used. LOH in tumours is indicated by arrows blood were quick frozen at –80°C. All relevant information about the tumours, e.g. size, node status, receptor status (oestrogen receptor (ER) and progesterone receptor (PR)), ploidy and S phase fraction, was recorded by the same department. In this study 204 invasive ductal breast tumours were used as controls for LOH analysis (Huiping et al, 1998).

### DNA isolation

Salting out procedure (Miller et al, 1988) and phenol extraction methods were used to obtain DNA from whole blood and tumour samples respectively.

### LOH determination

Microsatellite markers used for LOH analysis of chromosome 1p were: D1S233, D1S496, D1S209, D1S488, and D1S435. The microsatellite markers used for chromosome 3p were: D3S1211, D3S1029, D3S1217, D3S1210 and D3S1101. The markers used for 6q were: D6S262, D6S292, D6S409, D6S290 and D6S305. The markers used for 7q were: D7S518, D7S515, D7S523, D7S471 and D7S500. The markers used for 9p were: D9S156, D9S157, INFA, D9S171 and D9S104. The markers used for 11q were: D11S907, INT-2, D11S35, D11S4206, D11S925 and D11S921. The markers used for 13q were: D13S260, D13S171, D13S267, D13S219 and D13S263. The markers used for 16q were: D16S503, D16S496, D16S421, D16S545 and D16S512 for the 16q21–22.1 region containing the E-cadherin gene (Genome Database). The markers used for 17p were: D17S945, D17S921, D17S953, D17S925, D17S798 and D17S933. The markers used for 17q were: D17S800, D17S855, D17S1322, D17S579 and D17S784. The markers used for 18q were: D18S67, D18S474, D18S51, D18S70 and D18S61. The markers used for 20q were: D20S199, D20S118, D20S191, D20S119 and D20S196. The polymerase chain reaction (PCR) products were separated in an acrylamide sequencing gel and transferred to a positively charged nylon membrane, Hybond-N+ (Amersham, Aylesbury, UK) and baked for at least 2 h at 80°C. The non-radioactive detection method used to visualize the PCR products has been described previously (Vignal et al, 1993). Autoradiograms were inspected visually by at least two reviewers, comparing the intensity of alleles from normal and tumour DNA. The absence or a significant decrease of one allele in the tumour compared to the normal reference sample was considered as LOH.

### PCR–SSCP analysis

All 16 exons of E-cadherin gene were screened for inactivation mutations with a PCR–SSCP (single-strand conformation polymorphism) analysis on genomic DNA templates. The primers used in the SSCP analysis were described in Berx et al (1995*a*) and ordered from Pharmacia Biotech. Genomic DNA was used at 30 ng per 25 μl reaction mixture containing 5 pmol of the forward and reverse primers, 2.5 nmol of each dNTP, 0.5 units of DynaZyme polymerase. The samples were amplified in 35 cycles composed of 30 s of denaturation at 95°C, 60 s of annealing at 55–70°C, and finally 60 s of extension at 72°C. A hot start was used by adding the enzyme during the first cycle at about 72°C, after a preincubation time of 5 min at 94°C. A 4 μl aliquot of PCR products was mixed with 7 μl of formamide dye (95% formamide, 0.05% bromophenol blue and 0.05% xylene cyanol), denatured at 94°C for 10 min and snapcooled on ice. Aliquots of 2 μl were analysed simultaneously on two non-denaturing polyacrylamide gels (5% acrylamide with 2% cross-linking), either containing 5% glycerol or lacking glycerol. Electrophoresis was performed in 1 × TBE on vertical gels (390 × 330 × 0.4 mm) at 6w overnight or for 6 h at room temperature. The PCR products were visualized as the microsatellite markers.

### Sequencing of PCR products

Samples with abnormal mobility bands were amplified again for 35 cycles as described above. A 5 μl aliquot of the PCR product was then incubated with 10 U exonuclease I and 2 U shrimp alkaline phosphatase to remove excess of primers and dNTPs (US70995, Amersham). Sequences of both strands were determined by thermo

 *© 1999 Cancer Research Campaign*

**Table 1** LOH at different chromosome regions and immunohistochemical staining (IHCS) of E-cadherin in lobular breast tumours

| Tumour sample | 1 p | 3 p | 6 q | 7 q | 9 p | 11 q | 13 q | 16 q | 17 p | 17 q | 18 q | 20 q | E-cad IHCS |
|---|---|---|---|---|---|---|---|---|---|---|---|---|---|
| 402 | ND | + | ND | ND | ND | ND | ND | + | ND | ND | ND | ND | ND |
| 676 | + | – | – | – | – | – | – | + | ND | ND | ND | – | – |
| 743 | – | – | – | – | + | + | – | + | ND | + | – | + | ND |
| 745 | – | – | – | – | – | – | – | + | ND | ND | ND | – | ND |
| 806* | ND | ND | ND | ND | ND | ND | ND | + | ND | ND | ND | ND | – |
| 811 | – | – | – | + | – | + | + | + | – | ND | – | – | ND |
| 856 | – | – | – | – | – | – | – | + | ND | + | ND | – | + |
| 897 | + | + | – | – | – | + | – | + | – | + | + | + | ND |
| 908 | + | + | + | – | – | – | – | + | ND | ND | ND | – | ND |
| 936* | + | – | – | + | + | + | + | + | + | – | + | + | (+) |
| 986* | – | – | – | – | – | + | + | + | ND | – | ND | – | (+) |
| 1032 | – | – | ND | – | ND | – | – | + | ND | ND | ND | ND | (+) |
| 1036 | – | – | – | + | – | – | – | + | – | – | – | – | ND |
| 1061 | – | – | + | – | – | + | – | + | ND | – | ND | + | – |
| 1118 | – | – | + | – | – | – | + | + | ND | ND | ND | + | – |
| 1225 | – | ND | – | – | – | – | – | + | – | – | + | + | (+) |
| 1258 | – | – | – | – | – | – | + | + | – | – | – | + | – |
| 1301 | – | – | + | – | – | + | + | + | – | + | – | – | – |
| 1318* | – | – | – | + | – | + | + | + | – | – | + | – | – |
| 1327 | – | – | – | + | – | – | + | + | – | – | – | – | – |
| 1338 | + | – | – | + | – | – | + | + | + | + | – | + | – |
| 1341 | – | – | ND | + | ND | + | ND | + | ND | ND | ND | ND | – |
| 1367 | ND | ND | ND | ND | ND | ND | ND | + | ND | ND | ND | ND | ND |
| 1371 | ND | ND | ND | ND | ND | ND | ND | + | ND | ND | ND | ND | (+) |
| 1475 | – | – | – | + | – | + | – | ND | + | – | + | – | (+) |
| 1478 | ND | ND | ND | ND | ND | ND | ND | + | ND | ND | ND | ND | – |
| 1483 | + | – | – | – | – | – | – | + | + | – | – | – | – |
| 1499 | ND | ND | ND | ND | ND | ND | ND | ND | ND | ND | ND | ND | – |
| 1514 | – | – | – | – | – | – | – | + | – | – | – | – | + |
| 1515* | + | + | – | – | – | – | – | + | – | – | – | – | – |
| 1518 | – | + | + | + | – | + | + | + | + | – | – | + | – |
| 1520* | ND | ND | ND | ND | ND | ND | ND | ND | ND | ND | ND | ND | – |
| 1524 | + | + | – | + | + | – | – | ND | – | ND | – | – | – |
| 1534 | + | – | + | – | – | – | – | ND | + | + | ND | + | – |
| 1545 | – | – | – | – | + | – | – | ND | – | – | – | – | – |
| 1546 | ND | ND | ND | ND | ND | ND | ND | ND | ND | ND | ND | ND | – |
| 1548 | + | – | – | – | + | – | – | + | – | + | – | – | – |
| 1549 | + | – | + | + | – | + | – | ND | – | + | – | – | – |
| 1575 | – | – | + | + | – | – | – | ND | – | + | – | + | ND |
| 1583 | – | + | – | – | – | – | – | + | – | + | + | – | – |
| Useful | 11 | 7 | 8 | 12 | 5 | 12 | 10 | 31 | 6 | 10 | 6 | 11 | 29 |
| Total | 32 | 32 | 30 | 32 | 30 | 32 | 31 | 31 | 22 | 24 | 22 | 30 | 31 |
| % | 34 | 22 | 27 | 38 | 17 | 38 | 32 | 100 | 27 | 42 | 27 | 37 | 94 |

+, positive LOH or staining; –, negative LOH or staining; (+), weak staining; *, somatic mutation in the E-cadherin gene; ND, not determined sequenase DNA polymerase (Thermo Sequenase Radiolabeled Terminator Cycle Sequencing Kit, Amersham) using either one of the original PCR primers.

### Immunohistochemical staining

Immunohistochemistry was performed on 5-µm sections from paraffin-embedded tumour tissue blocks with monoclonal antibody HECD-1 (Zymed Laboratories, South San Francisco, CA, USA) using the antigen retrieval protocol described by Hazelbag et al (1995).

### Statistical analysis

A $\chi^2$ test was used to assess the relationship between LOH in lobular tumours and LOH in ductal tumours at 1p, 3p, 6q, 7q, 9p, 11q, 13q, 16q, 17q, 18q and 20q. We also analysed the association of LOH at different chromosome regions with the categorized prognostic variables, LOH at other chromosome regions and E-cadherin gene mutations by $\chi^2$ test and Fisher's exact test.

### RESULTS

### LOH analysis

LOH by the use of polymorphic microsatellite markers at chromosomes 1p, 3p, 6q, 7q, 9p, 11q, 13q, 16q, 17p, 17q, 18q and 20q in 40 lobular tumours was analysed (Figure 1). LOH for these chromosome regions ranged from 17% to 100% (Tables 1 and 2). The highest percentage of LOH was detected at the 16q21–q22.1 region where all examined cases (31/31) were positive. Table 2 shows the $\chi^2$ analysis comparing LOH in lobular tumours with LOH in ductal tumours investigated in our laboratory at the different chromosome regions. There was a significant association

*© 1999 Cancer Research Campaign* 

**Table 2** Chi-square analysis comparing loss of heterozygosity (LOH) at different chromosome regions of tumour DNA to histological types

| Chromosome region | Histological type | LOH/total | % | *P* |
|---|---|---|---|---|
| 1 p | Lobular | 11/32 | 34% | |
| | Ductal | 111/194 | 57% | 0.02[c] |
| 3 p | Lobular | 7/32 | 22% | |
| | Ductal | 48/96 | 50% | 0.0054[b] |
| 6 q | Lobular | 8/30 | 27% | |
| | Ductal | 50/112 | 45% | 0.08 |
| 7 q | Lobular | 12/32 | 38% | |
| | Ductal | 54/180 | 30% | 0.40 |
| 9 p | Lobular | 5/30 | 17% | |
| | Ductal | 60/134 | 45% | 0.0044[b] |
| 11 q | Lobular | 12/32 | 38% | |
| | Ductal | 62/107 | 58% | 0.04[c] |
| 13 q | Lobular | 10/31 | 32% | |
| | Ductal | 88/146 | 60% | 0.0044[b] |
| 16 q | Lobular | 31/31 | 100% | |
| | Ductal | 57/78 | 73% | <0.001[a] |
| 17 q | Lobular | 10/24 | 42% | |
| | Ductal | 28/46 | 61% | 0.13 |
| 18 q | Lobular | 6/22 | 27% | |
| | Ductal | 123/192 | 64% | <0.001[a] |
| 20 q | Lobular | 11/30 | 37% | |
| | Ductal | 39/117 | 33% | 0.73 |

[a]99.9% confidence interval. [b]99% confidence interval. [c]95% confidence interval.

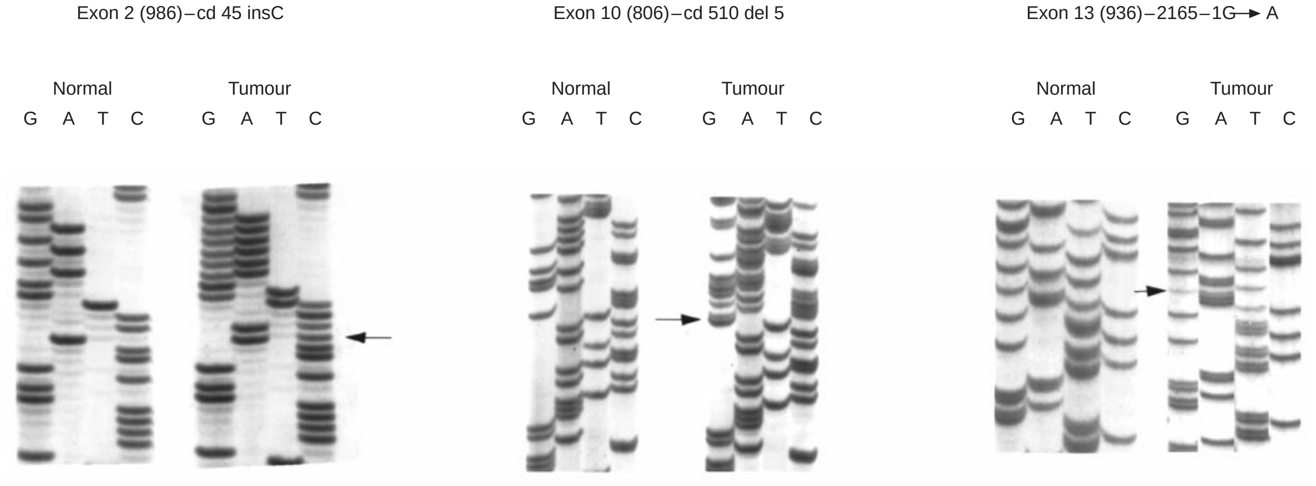

**Figure 2** Three examples of sequence analysis of abnormally shifted PCR–SSCP bands, yielding three different mutation types as indicated at the bottom. The arrows indicate the following sequence changes: (**A**) one basepair insertion cd 45 ins C; (**B**) five basepair deletion cd 510 del cccag; (**C**) splice site mutation 2165–1 G→A. The sample numbers are in parentheses between lobular tumours and LOH at 16q (99.9% confidence interval). There was also a significant association between ductal tumours and LOH at 1p, 3p, 9p, 11q, 13q and 18q (Table 2). By comparing all investigated chromosome regions in lobular breast tumours, a significant association was detected between LOH at 13q and LOH at 7q ($P = 0.049$) and between LOH at 13q and LOH at 11q ($P = 0.049$).

### Mutational analysis of 40 lobular breast cancers by PCR–SSCP

We studied a set of breast cancers from 40 patients for occurrence of E-cadherin mutations. Mobility shifts were found in the amplicons 1, 2, 3, 4–5, 10, 13, 14 and 16 (Table 3). Tumours with mutation in the E-cadherin gene, 936, 986, 1318 and 1515, show LOH at several chromosomes in addition to chromosome 16q: chromosome 1p, 7q, 9p, 11q, 13q, 17p, 18q and 20q; chromosome 11q and 13q; chromosome 7q, 11q, 13q and 18q; and chromosome 1p and 3p respectively (Table 1).

### DNA sequence analysis

By direct sequence analysis of the PCR products, five truncation mutations were identified among the 40 lobular tumours (Figure 2 and Table 3). Three frameshift insertions and two frameshift deletions were found, all resulting in premature downstream stop

 *© 1999 Cancer Research Campaign*

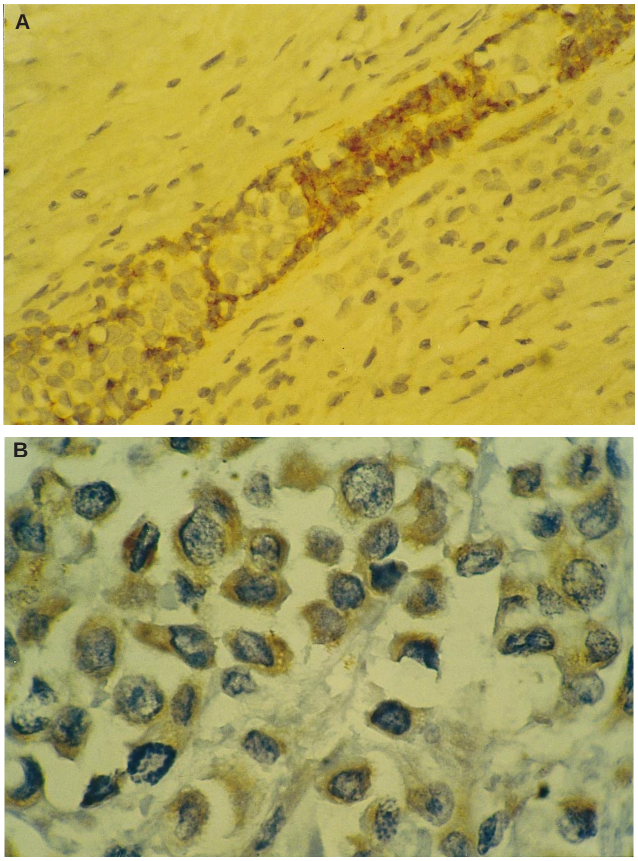

**Figure 3** (**A**) Case no. 1338. Normal duct with positive staining for E-cadherin in non-neoplastic ductal cells and lobular carcinoma in situ within the duct negative for E-cadherin. In the surrounding tissue infiltrating lobular carcinoma cells are negative for E-cadherin. (**B**) Case no. 936. Infiltrating lobular carcinoma cells showing mild diffuse cytoplasmic staining for E-cadherin codons (Table 3). Most of the insertions and deletions were rather small, causing 1-bp to 5-bp changes. Only one larger deletion of 22 bp was identified. We also identified one putative splice site mutation for exon 13 (Figure 2 and Table 3). In the mutated splice acceptor sequence of exon 13, GT was converted by a single base substitution to AT (Figure 2 and Table 3). All of the six mutations were found only once and have not been previously reported. Sequencing of DNA for blood from all six patients with mutations in breast tumours indicates that the mutations are not germline, but tumour-specific.

Besides the six mutations, we also identified nine different polymorphisms of which four have been described before (Risinger et al, 1994; Berx et al, 1995*a*, 1996). Besides the frequent polymorphism in the intron 4, a previously unreported but frequent polymorphism (four out of 38) was identified in the intron 15 sequence from amplicon 16. Polymorphisms are shown in Table 3.

### Immunohistochemistry

Of the 31 lobular tumours analysed by immunohistochemistry, 29 (94%) showed no or reduced membrane-associated E-cadherin staining with monoclonal antibody HECD-1 and only two showed a clear membrane-associated expression of E-cadherin (Figure 3 and Table 1). In particular, four samples 806, 986, 1318 and 1515 with truncation mutations plus LOH at 16q22.1 were negative or showed reduced E-cadherin protein expression (Table 3 and Figure 3). All lobular breast tumours lack membrane staining, while two of them show weak cytoplasmic staining.

### Association analysis of the lobular breast tumours

We found an association between E-cadherin gene mutations and LOH at 13q and 16q (*P* = 0.05). A significant association was also found between LOH at 3p and high S phase, LOH at 9 p and low ER and PR content, LOH at 17p and aneuploidy (*P* = 0.033, 0.011, 0.001, 0.014 respectively). A trend was detected between LOH at 1p and tumours larger than 2 cm (*P* = 0.052).

### DISCUSSION

In this study, LOH at 16q22.1 was detected in all lobular breast tumours examined. This is a higher rate than those reported in previous studies (Tsuda et al, 1994; Skirnisdottir et al, 1995; Berx et al, 1996) presumably due to difference in the set of markers used. LOH at 16 q in lobular tumours was compared with LOH at 16 q in ductal tumours using the same microsatellite markers. The detected association suggests that the chromosome 16q22.1 region contains one or more suppressor genes particularly relevant to lobular breast carcinogenesis. LOH for the 16q22.1 region has been detected in several carcinoma types besides breast cancer, also suggesting the presence of tumour suppressor genes at this region (Tsuda et al, 1990; Bergerheim et al, 1991; Sato et al, 1991). We also compared LOH at other chromosome regions in lobular and ductal breast tumours and found a higher LOH at 1 p, 3 p, 9 p, 11 q, 13 q and 18 q in ductal tumours, indicating a difference in genetic alterations in the two histological types of breast cancers.

The human E-cadherin gene has been mapped to chromosome 16q22.1 (Mansouri et al, 1988; Natt et al, 1989). Its expression was reduced in several types of human carcinomas (Shimoyama et al, 1991; Bussemakers et al, 1992; Inoue et al, 1992; Umbas et al, 1992). Mutations of the E-cadherin gene have been identified in human tumours and tumour cell lines (Becker et al, 1993, 1994; Kanai et al, 1994; Oda et al, 1994; Risinger et al, 1994; Berx et al, 1995*a*, 1996). These observations in combination with LOH at 16q22.1 indicate that the E-cadherin gene is a tumour suppressor gene. Our data reported here consolidate the evidence for E-cadherin playing an important role as a typical tumour suppressor in lobular breast carcinomas. Since no mutations in the E-cadherin gene have been detected in tumours in the breast of the ductal histological type (Berx et al, 1996), this provides the clearest evidence of molecular difference in the two main histological types of breast carcinomas. Nonetheless, reduced expression of E-cadherin has been found in both lobular and ductal breast cancers (Oka et al, 1993; Gamallo et al, 1996) and detection of chromosome 16q22.1 is the highest documental loss of a chromosome region in sporadic breast cancers of both histological types (Skirnisdottir et al, 1995; this study). Two explanations of this difference are possible, either: (1) a gene other than the E-cadherin gene is the target of the 16q22.1 deletion in ductal compared to lobular carcinomas of breast cancer; or (2) the progression of ductal carcinomas is more sensitive to loss of one copy of the E-cadherin gene, and corresponding reduction of expression, than lobular breast carcinomas, where both copies need to be eliminated for further progression to malignant invasive growth. The LOH detected in this study is probably due to loss of genetic material for chromosome 16q22.1 since

© 1999 Cancer Research Campaign

**Table 3** Summary of E-cadherin gene mutations and polymorphisms detected in a series of 40 lobular breast tumours

| Amplicon No.[a] | Tumour sample | Mutation site[b] | Mutation | Nucleotide change | LOH[c] | E-cad IHC[d] |
|---|---|---|---|---|---|---|
| 1 | 1318 | cd 8 | frameshift | del 22 (stop at cd 48)[e] | yes | – |
| 2 | 986 | cd 45 | frameshift | ins C (stop at cd 58) | yes | (+) |
| 2 | 1520 | cd 51 | frameshift | ins G (stop at cd 58) | NT | – |
| 10 | 806 | cd 510 | frameshift | del 5 (stop at cd 534)[f] | yes | – |
| 13 | 936 | intron 13 | splice site | 2165–1 G→A | yes | (+) |
| 14 | 1515 | cd 726 | frameshift | ins T (stop at cd 747) | yes | – |

| Amplicon | Site | Polymorphism | Observed frequency |
|---|---|---|---|
| 3 | intron 2 | tttctccc→tttctgccc | 2/38 |
| 3 | cd 115 | ACG(Thr)→ACA(Thr) | 1/38[g] |
| 4–5 | intron 4 | gaaac→gaaag | 6/38[h] |
| 13 | cd 692 | GCC(Ala)→GCT(Ala) | 3/38[i] |
| 14 | cd 751 | AAC(Asn)→AAT(Asn) | 1/38[j] |
| 14 | cd 764 | GAC(Asp)→GAT(Asp) | 1/38 |
| 16 | cd 878 | GGC(Gly)→GGT(Gly) | 1/38 |
| 16 | intron 15 | ttgag→tttag | 4/38 |
| 16 | intron 15 | ttctt→ttgtt | 1/38 |

[a]Amplicons contain besides exons also flanking intronic sequences and in the case of amplicon 4–5 the whole intron 4 is present, too. [b]cd, codon. [c]LOH, loss of heterozygosity at 16q22.1. [d]E-cad IHC: E-cadherin specific immunohistochemistry. NT, no tumour material available for LOH analysis. –, no cell surface associated staining. (+), weak or cytoplasmic staining, +, normal staining. [e]22 bp deletion at codon 8 of exon 1: 5′-ctctcggcgctgctgctgctgc-3′. [f]5 bp deletion at codon 510 of exon 10: 5′-cccag-3′. [g–j]These polymorphisms were also reported by Berx et al. (1995a). [i]This polymorphism was also reported by Risinger et al (1994). [j]This polymorphism was reported previously by Risinger et al (1994) and by Becker et al (1994).

reduced expression of the E-cadherin is detected as well, but our method in detecting LOH does not exclude a duplication of a locus due to mitotic recombination.

Altogether, we examined 40 lobular breast tumours on the genomic level for E-cadherin mutations. Besides polymorphisms, six mutations were identified by our E-cadherin PCR/SSCP analysis. None of the mutations described here have been reported before. In agreement with the mutations reported by Berx et al (1996), most of the mutations (5/6) were caused by frameshifts, usually resulting in premature stop codons (Table 3). Although a large series of lobular tumours was analysed, we could not identify any non-sense mutation, in contrast with a previous report on five non-sense mutations in a series of 48 lobular tumours (Berx et al, 1995*a*, 1996). Besides truncation mutations we also found a splice site mutation, affecting the acceptor splice site and probably causing skipping of exon 14 (Table 3). This particular tumour shows a weak cytoplasmic E-cadherin staining. Becker et al (1993, 1994) have found frequent skipping of exon 8 or 9 in diffuse gastric carcinomas. It is noteworthy that exon 14 is encoding the part of the transmembrane domain necessary for binding membrane and the part of the cytoplasmic domain necessary for binding β-catenin and plakoglobin (γ-catenin) (Aberle et al, 1994). E-cadherins are linked to the actin cytoskeleton by the introduction of catenins, resulting in tight cell–cell adhesion (Cowin, 1994). The aberrant transmembrane domain and cytoplasmic domain can affect the cell–cell adhesion. Interestingly, the six novel mutations were scattered over all five E-cadherin domains, including the signal and precursor sequences (Table 3).

The various truncated E-cadherin peptides are likely to be secreted and to have some residual function which could interfere with proper cell–cell adhesion in the peritumoral tissues (Berx et al, 1996). Furthermore, these fragments and even small deca-peptides containing the HAV sequence, known to be involved in homophilic recognition, can disturb proper cell–cell adhesion (Blaschuk et al, 1990). Therefore, it may be interesting to investigate the treatment of lobular tumours with related antibodies that can be synthesized in vitro according to the genes with truncation or splice site mutations.

For all informative lobular breast tumours, which we found to lack membrane E-cadherin expression, we identified both LOH of 16q22.1 harbouring the E-cadherin locus and mutations in the remaining allele, in accordance with the classical two-hit theory of Knudson for tumour suppressor genes (Knudson, 1985). This supports the results reported by Berx et al (1995*a*, 1996). This is also supported by an association between E-cadherin gene mutations and LOH at 16q. Presumably, LOH at 13q could enhance the lobular tumour growth triggered by E-cadherin gene mutation and LOH at 16q because we also found an association between E-cadherin gene mutations and LOH at 13q. Transfection of MET/6 mouse mammary carcinoma cells, which lack E-cadherin, with an exogenous E-cadherin expression vector, resulted in tighter adhesion of multicellular spheroids and a reduced proliferative fraction in three-dimensional culture (St Croix et al, 1998). Exposure to E-cadherin-neutralizing antibodies in three-dimensional culture simultaneously prevented adhesion and stimulated proliferation of E-cadherin transfectants as well as a panel of human colon, breast and lung carcinoma cell lines that express functional E-cadherin (St Croix et al, 1998). From these, it can be concluded that E-cadherin, classically described as an invasion suppressor, is also a major growth suppressor.

We identified loss of or reduced E-cadherin expression in 29 out of 31 lobular breast tumours, in accordance with earlier studies (Gamallo et al, 1993; Rasbridge et al, 1993). However, only six out of 29 lobular tumours lacking E-cadherin expression showed detectable E-cadherin mutations, suggesting that loss of one copy of the E-cadherin gene may affect the expression of the remaining allele. This is lower frequency of mutations than reported by Berx et al (1995*a*, 1996). The number of mutations reported here is

 *© 1999 Cancer Research Campaign*

probably underestimated, either due to low efficiency of SSCP or that some tumours were lacking data from all exons. In addition to this the reduced expression may be due to transcriptional defects (Ji et al, 1997). Synthesis of mRNA could be affected by mutations in the promoter region, or in intron located regulatory sequences, whereas stability of mRNA could be affected by mutations in the untranslated regions (Berx et al, 1996). CpG-island overspanning intron 1 of the E-cadherin gene (Berx et al, 1995*b*) was found to be densely methylated in breast cancer cell lines, and this correlated with loss of E-cadherin expression (Graff et al, 1995). Moreover, p53 protein accumulation and c-erbB-2 protein overexpression may play a role in regulation of E-cadherin expression (Bukholm et al, 1997).

Catenins link E-cadherin molecules to the actin cytoskeleton and lay a solid foundation for the tight cell–cell adhesion (Cowin, 1994). Therefore, loss of or reduced catenin expression also interferes with cell–cell adhesion. Simultaneous loss of E-cadherins and catenins have been detected in invasive lobular breast cancer and lobular carcinoma in situ (De Leeuw et al, 1997). Thus, it may be important to detect mutations in the catenin genes in lobular breast tumours or other epithelial carcinomas.

A number of studies have revealed that chromosome deletions in breast cancers may occur in preferred combinations with respect to growth advantage, e.g. 1p and 3p (Ragnarsson et al, 1996), 7q and 1p (Kristjansson et al, 1997), 3p and 6q (Bragadottir et al, 1995), 9p and 6q (Eiriksdottir et al, 1995), 13q and 17p (Anderson et al, 1992), 11p and 17p (Takita et al, 1992), 18q and 1p, 7q, 9p, 13q and 17q (Huiping et al, 1998). In order to determine whether there are specific combinations of chromosome deletions in lobular breast tumours, we compared LOH at different chromosome regions and found a weak association between LOH at 13q and LOH at 7q and 11q.

LOH at different chromosome regions was compared with various clinicopathological variables of the lobular breast carcinomas. A significant association was found between LOH at 9p and low ER and low PR. This suggests that LOH at 9p could be involved in the loss of ER and PR content. A weak association was found between LOH at 3p and high S phase fractions and a trend between LOH at 1p and tumour larger than 2 cm. This suggests that genes at 3p and 1p could possibly have a restraining effect on the rate of cell proliferation, and the loss of them would lead to a rapid growth. We also found a weak association between LOH at 17p and aneuploidy, indicating that LOH at 17p could be associated with an unstable genome.

### ACKNOWLEDGEMENTS

The authors wish to thank Kristrun Olafsdottir and Sigrun Kristjansdottir for help with the immunohistochemical staining. This work was financially supported by the Icelandic Research Council and the Icelandic Cancer Society.

### REFERENCES

Aberle H, Butz S, Stappert J, Weissig H, Kemler R and Hoschuetzky H (1994) Assembly of the cadherin–catenin complex in vitro with recombinant proteins. *J Cell Sci* **107**: 3655–3663

Anderson TI, Gaustad A, Farrants GW, Nesland JM, Tweit KM and Borresen AL (1992) Genetic alterations of tumour suppressor gene regions 3 p, 11 p, 13 q, 17 p and 17 q in human breast carcinoma. *Genes Chromosomes Cancer* **4**: 113–121

Becker KF, Atkinson MJ, Reich U, Huang HH, Nekards H, Siewert JR and Hofler H (1993) Exon skipping in the E-cadherin gene transcript in metastatic human gastric carcinomas. *Hum Mol Genet* **2**: 803–804

Becker KF, Atkinson MJ, Reich U, Becker J, Nekarda H, Siewert JR and Hofler H (1994) E-cadherin gene mutations provide clues to diffuse type gastric carcinomas. *Cancer Res* **54**: 3845–3852

Bergerheim US, Kunimi K, Collins VP and Ekman P (1991) Deletion mapping of chromosome 8, 10, and 16 in human prostatic carcinoma. *Genes Chromosomes Cancer* **3**: 215–220

Berx G, Cleton-Jansen AM, Nollet F, de Leeuw WJF, van de Vijver MJ, Cornelisse C and van Roy F (1995*a*) E-cadherin is a tumour/invasion suppressor gene mutated in human lobular breast cancers. *EMBO J* **14**: 6107–6115

Berx G, Staes K, van Hengel J, Molemans F, Bussemakers MJG, van Bokhoven A and van Roy F (1995*b*) Cloning and characterization of the human invasion suppressor gene E-cadherin (CDH1). *Genomics* **26**: 281–289

Berx G, Cleton-Jansen AM, Strumane K, de Leeuw WJF, Nollet F, van Roy F and Cornelisse C (1996) E-cadherin is inactivated in a majority of invasive human lobular breast cancers by truncation mutations throughout its extracellular domain. *Oncogene* **13**: 1919–1925

Blaschuk OW, Sullivan R, David S and Pouliot Y (1990) Identification of a cadherin cell adhesion recognition sequence. *Dev Biol* **139**: 227–229

Bragadottir G, Eiriksdottir G, Sigurdsson A, Barkardottir RB, Gudmundsson J, Jonasson JG and Ingvarsson S (1995) Loss of heterozygosity at chromosome 6q correlates with tumour progression and patient survival. *Int J Oncol* **7**: 871–876

Bukholm IK, Nesland JM, Karesen R, Jacobsen U and Borresen-Dale AL (1997) Expression of E-cadherin and its relation to the p53 protein status in human breast carcinomas. *Virchows Arch* **431**: 317–321

Bussemakers MJG, van Moorselaar RJA, Giroldi LA, Ichikawa T, Isaacs JT, Takeichi M, Debruyne FMJ and Jack AS (1992) Decreased expression of E-cadherin in the progression of rat prostatic cancer. *Cancer Res* **52**: 2916–2922

Cleton-Jansen AM, Moerland EW, Kuipers-Dijkshoorn NJ, Callen DF, Sutherland GR, Hansen B, Devilee P, Cornelisse CJ (1994) At least two different regions are involved in allelic imbalance on chromosome arm 16 q in breast cancer. *Gene Chromosomes Cancer* **9**: 101–107

Cowin P (1994) Unraveling the cytoplasmic interactions of the cadherin superfamily. *Proc Natl Acad Sci USA* **91**: 10759–10761

De Leeuw WJ, Berx G, Vos CB, Peterse JL, Van de Vijver MJ, Litvinov S, Van Roy F, Cornelisse CJ and Cleton-Jansen AM (1997) Simultaneous loss of E-cadherin and catenin in invasive lobular breast cancer and lobular carcinoma in situ. *J Pathol* **183**: 404–411

Eiriksdottir G, Sigurdsson A, Jonasson JG, Agnarsson BA, Sigurdsson H, Gudmundsson J, Bergthorsson JT, Barkardottir RB, Egilsson V and Ingvarsson S (1995) Loss of heterozygosity on chromosome 9 in human breast cancer: association with clinical variables and genetic changes at other chromosome regions. *Int J Cancer* **64**: 378–382

Frixen UH, Behrens J, Sachs M, Eberle G, Voss B, Warda A, Lochner D and Birchmeier W (1991) E-cadherin-mediated cell–cell adhesion prevents invasiveness of human carcinoma cells. *J Cell Biol* **113**: 173–185

Gamallo C, Palacios J, Suarez A, Pizarro A, Navarro P, Quintanilla M and Cano A (1993) Correlation of E-cadherin expression with differentiation grade and histological type in breast carcinomas. *Am J Pathol* **142**: 987–993

Gamallo C, Palacios J, Benito N, Limeres MA, Suarez A, Pastrana F, Cano A and Calero F (1996) Expression of E-cadherin in 230 infiltrating ductal breast carcinomas – relationship to clinicopathological features. *Int J Oncol* **9**: 1207–1212

Gayther SA, Gorringe KL, Ramus SJ, Huntsman D, Roviello F, Grehan N, Machado JC, Pinto E, Seruca R, Halling K, MacLeod P, Powell SM, Jackson CE, Ponder BA and Caldas C (1998) Identification of germ-line E-cadherin mutations in gastric cancer families of European origin. *Cancer Res* **58**: 4086–4089

Graff JR, Herman JG, Lapidus RG, Chopra H, Xu R, Jarrard DF, Isaacs WB, Pitha PM, Davidson NE and Baylin SB (1995) E-cadherin expression is silenced by DNA hypermethylation in human breast and prostate carcinomas. *Cancer Res* **55**: 5195–5199

Guilford P, Hopkins J, Harrsway J, Mcleod M, Mcleod N, Harawira P, Taite H, Scoular R, Miller A and Reeve AE (1998) E-cadherin germline mutations in familial gastric cancer. *Nature* **392**: 402–405

Gumbiner B, Stevenson B and Grimaldi A (1988) The role of the cell adhesion molecule uvomorulin in the formation and maintenance of the epithelial junctional complex. *J Cell Biol* **107**: 1575–1587

Hazelbag HM, Van de Brock LJCM, Van Dorst EBL, Offerhaus GJA, Fleuren GJ and Hogendoorn PCW (1995) Immunostaining of chain-specific keratins on formalin-fixed, paraffin-embedded tissues: a comparison of various antigen

© 1999 Cancer Research Campaign retrieval systems using microwave heating and proteolytic pretreatments. *J Histochem Cytochem* **43**: 429–437
Hiraguri S, Godfrey T, Nakamura H, Graff J, Collins C, Shayesteh L, Doggett N, Johnson K, Wheelock M, Herman J, Baylin S, Pinkel D and Gray J (1998) Mechanisms of inactivation of E-cadherin in breast cancer cell lines. *Cancer Res* **58**: 1972–1977
Huiping C, Eiriksdottir G, Sigurdsson A, Sigurgeirsdottir JR, Barkardottir RB, Egilsson V and Ingvarsson S (1998) High frequency of LOH at chromosome 18 q in human breast cancer: association with high S-phase fraction and low progesterone receptor content. *Anticancer Res* **18**: 1031–1036
Ingvarsson S (1999) Molecular genetics of breast cancer progression. *Sem Cancer Biol* **9**: 277–288
Inoue M, Ogawa H, Miyata M, Shiozaki H and Tanizawa O (1992) Expression of E-cadherin in normal, benign, and malignant tissues of female genital organs. *Am J Clin Pathol* **98**: 76–80
Ji X, Woodard AS, Rimm DL and Fearon ER (1997) Transcriptional defects underlie loss of E-cadherin expression in breast cancer. *Cell Growth Diff* **8**: 773–778
Kanai Y, Oda T, Tsuda H, Ochiai A and Hirohashi S (1994) Point mutation of the E-cadherin gene in invasive lobular carcinoma of the breast. *Jpn J Cancer Res* **85**: 1035–1039
Knudson AG (1985) Hereditary cancer, oncogenes, and antioncogenes. *Cancer Res* **45**: 1437–1443
Kristjansson AK, Eiriksdottir RB, Ragnarsson G, Sigurdsson A, Gudmundsson J, Barkardottir RB, Jonasson JG, Egilsson V and Ingvarsson S (1997) Loss of heterozygosity at chromosome 7 q in human breast cancer: association with clinical variables. *Anticancer Res* **17**: 93–98
Mansouri A, Spurr N, Goodfellow PN and Kemler R (1988) Characterization and chromosomal localization of the gene encoding the human cell adhesion molecule uvomorulin. *Differentiation* **38**: 67–71
Miller SA, Dykes DD and Polesky HF (1988) A simple salting out procedure for extracting DNA from human nucleated cells. *Nucleic Acids Res* **16**: 1215
Natt E, Magenis RE, Zimmer J, Mansouri A and Scherer G (1989) Regional assignment of the human loci for uvomorulin (UVO) and chymotrypsinogen B (CTRB) with the help of two overlapping deletions on the long arm of chromosome 16. *Cytogenet Cell Genet* **50**: 145–148
Navarro P, Gomez M, Pizarro A, Gamallo C, Quintanilla M and Cano A (1991) A role for the E-cadherin cell–cell adhesion molecule during tumour progression of mouse epidermal carcinogenesis. *J Cell Biol* **115**: 517–533
Oda T, Kanai Y, Oyama T, Yoshiura K, Shimoyama Y, Birchmeier W, Sugimura T and Hirohashi S (1994) E-cadherin gene mutations in human gastric carcinoma cell lines. *Proc Natl Acad Sci USA* **91**: 1858–1862
Ragnarsson G, Sigurdsson A, Eiriksdottir G, Barkardottir RB, Jonasson JG and Ingvarsson S (1996) Loss of heterozygosity at chromosome 1 p in human breast cancer: Association with high S-phase, reduced patient survival and deletion at other chromosomal regions. *Int J Oncol* **9**: 731–736
Rasbridge SA, Gillett CE, Sampson SA, Walsh FS and Millis RR (1993) Epithelial (E-) and placental (P-) cadherin cell adhesion molecule expression in breast carcinoma. *J Pathol* **169**: 245–250
Risinger JI, Berchuck A, Kohler MF and Boyd J (1994) Mutations of the E-cadherin gene in human gynecologic cancers. *Nature Genet* **7**: 98–102
Sato T, Saito H, Morita R, Koi S, Lee JH and Nakamura Y (1991) Allelotype of human ovarian cancer. *Cancer Res* **51**: 5118–5122
Shapiro L, Fannon AM, Kwong PD, Thompson A, Lehmann MS, Grubel G, Legrand JF, Als-Nielsen J, Colman DR and Hendrickson WA (1995) Structural basis of cell–cell adhesion by cadherins. *Nature* **374**: 327–337
Shimoyama Y and Hirohashi S (1991) Expression of E- and P-cadherin in gastric carcinomas. *Cancer Res* **51**: 2185–2192
Siitonen SM, Kononen JT, Helin HJ, Rantala IS, Holli KA and Isola JJ (1996) Reduced E-cadherin expression is associated with invasiveness and unfavorable prognosis in breast cancer. *Am J Clin Pathol* **105**: 394–402
Skirnisdottir S, Eiriksdottir G, Baldursson T, Barkardottir RB, Egilsson V, Ingvarsson S (1995) High frequency of allelic imbalance at chromosome region 16q22–23 in human breast cancer: correlation with high PgR and low S phase. *Int J Cancer* **64**: 112–116
St Croix B, Sheehan C, Rak JW, Florenes VA, Slingerland JM and Kerbel RS (1998) E-cadherin-dependent growth suppression is mediated by the cyclin-dependent kinase inhibitor p27 (KIP1). *J Cell Biol* **142**: 557–571
Takeichi M (1990) Cadherins: a molecular family important in selective cell–cell adhesion. *Annu Rev Biochem* **59**: 237–252
Takita KI, Sato T, Migyagi M, Watatani M, Akiyama F, Sakamoto G, Kasumi F, Abe R and Nakamura Y (1992) Correlation of loss of alleles on the short arm of chromosome 11 and 17 with metastasis of primary breast cancer to lymph nodes. *Cancer Res* **52**: 3914–3917
Tsuda H, Zhang W, Shimosato Y, Yokota J, Terada M, Sugimura T, Miyamura T and Hirohashi S (1990) Allele loss on chromosome 16 associated with progression of human hepatocellular carcinoma. *Proc Natl Acad Sci USA* **87**: 6791–6794
Tsuda H, Callen DF, Fukutomi T, Nakamura Y, Hirohashi S (1994) Allele loss on chromosome 16q24.2-qter occurs frequently in breast cancer irrespectively of differences in phenotype and extent of spread. *Cancer Res* **54**: 513–517
Umbas R, Schalken J, Aalders TW, Carter BS, Karthaus HFM, Schaafsma HE, Debruyne FMJ and Isaacs WB (1992) Expression of the cellular adhesion molecule E-cadherin is reduced or absent in high-grade prostate cancer. *Cancer Res* **52**: 5104–5109
Vignal A, Gyapay G, Hazan J, Nguyen S, Dupraz C and Cheron N (1993) Nonradioactive multiplex procedure for genotyping of microsatellite markers. Methods in Molecular Genetics, Vol. 1. San Diego: Academic Press Inc, pp 211–221

 *© 1999 Cancer Research Campaign*
